# Arguments for and against self and non-self root recognition in plants

**DOI:** 10.3389/fpls.2014.00614

**Published:** 2014-11-06

**Authors:** Stephen Depuydt

**Affiliations:** ^1^Ghent University Global Campus, Incheon, South Korea; ^2^Department of Plant Biotechnology and Bioinformatics, Ghent University, Ghent, Belgium; ^3^Department of Plant Systems Biology, Flanders Institute for Biotechnology, Ghent, Belgium

**Keywords:** root–root interaction, root competition, identity recognition, self/non-self recognition, root growth

## Abstract

Root–root interaction research gained more and more attention over the past few years. Roots are pivotal for plant survival because they ensure uptake of water and nutrients. Therefore, detection of adjacent roots might lead to competitive advantages. Several lines of experimental evidence suggest that roots have ways to discriminate non-related roots, kin, and—importantly—that they can sense self/non-self roots to avoid intra-plant competition. In this mini-review, the existence of self/non-self recognition in plant roots will be discussed and the current knowledge on the mechanisms that could be involved will be summarized. Although the process of identity recognition is still not completely understood, interesting data are available and emerging new technologies will certainly aid to better understand this research field that can have an important biological, ecological, and agricultural impact.

## INTRODUCTION

Competition among coexisting plants—most restrictively defined as a negative interaction among individuals with reduced growth, survival, or fecundity of neighbors as a consequence (Casper and Jackson, 1997)—is all about the availability of space, nutrients, water, and light. This contest is thought to be, at least in part, responsible for the plant diversity in different ecosystems (Goldberg and Barton, 1992; Wilson and Tilman, 1993). Moreover, it is, if anything, a showcase for the remarkable adaptive plasticity of plants, i.e., their ability to alter their morphology and physiology in response to environmental stimuli (Bradshaw, 1965; reviewed in Hodge, 2009; Ford, 2014).

Roots are pivotal for plant survival because they ensure the uptake of nutrients and water and they secure fixation in the soil; hence, the growing interest in the study of belowground plant competition. Plants that grow together in one soil volume depend on the same resources and rearrange their root systems to gain access to these limited supplies (Robinson, 1994). Indeed, root systems develop differently when neighboring roots are present and their growth responses vary. These responses are determined by species, relatedness, even genotype, and by self or non-self identity of the competing roots (reviewed in Schenk et al., 1999; Chen et al., 2012). The latter indicate interactions among roots of the same individual plant (“self”) or of different plants (“non-self”), whereby plants also seem to be able to recognize kin (Dudley and File, 2007). Thus, roots possess a so-called “identity recognition.” However, how do roots recognize other roots? Although root–root interaction studies are extremely complex due to the many factors that influence root competition and the inaccessibility of the belowground root system, recent efforts addressed this still open question. Nevertheless, the exploration of molecular mechanisms of root identity recognition is limited. With next generation sequencing methods becoming more available in research practices, it seems only timely to address this question by using such state of the art techniques, for which proteomics and metabolomics approaches could also prove useful.

Here, knowledge on root–root dynamics between interacting plants will be summarized and new advances will be discussed that cannot only enhance the understanding of plant evolution and biology, but can also have an impact on ecology and agriculture.

## ROOT–ROOT INTERACTIONS: HOW THE ROOT SYSTEM RESPONDS TO NEIGHBORING ROOTS

**FIGURE 1:**
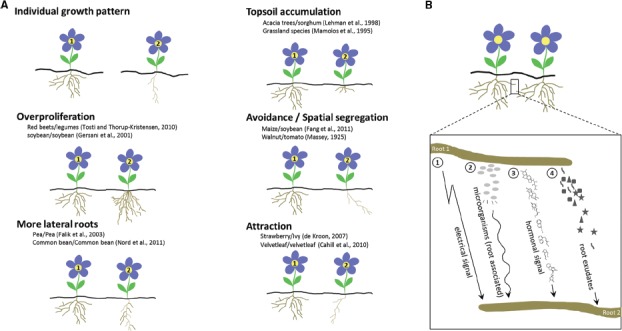
**Figure 1. Common root responses to neighboring plants and possible mechanisms of root identity recognition. (A)** Range of root responses toward neighboring roots. Two plants are depicted (plant 1 and 2) of which plant 2 shows a differential root response because it is neighbored with plant 1. Plants 1 and 2 can be either of the same species or of different species. Examples (both intra- and interspecifically) of interacting species, are provided for each response: overproliferation of the root system, i.e., root biomass changes which may include main root length increases, more adventitious rooting, more and higher order lateral roots, etc.; increased lateral rooting; accumulation in the top soil; spatial segregation; root attraction. **(B)** Four proposed mechanisms in self/non-self root recognition: resonant amplification of electrical or hormonal signals (Schenk et al., 1999; Falik et al., 2003); root associated microorganisms and/or their secreted substances (Steenhoudt and Vanderleyden, 2000); and soluble metabolites in root exudates (Biedrzycki et al., 2010; Caffaro et al., 2011; Fang et al., 2013; Semchenko et al., 2014). A non-self root–root interaction is shown.

A lot of experimental evidence suggests that plants alter their root growth in the presence of other plants (for a review, see Schenk et al., 1999). Pioneering work on root interaction focused mainly on spatial segregation, such as intraspecifically in *Parthenium argentatum* (guayule; Muller, 1946) or *Prunus persica* (peach) trees (Bini and Chisci, 1961), or interspecifically, such as *Juglans nigra* (black walnut) roots that exclude *Solanum lycopersicum* (tomato) roots (Massey, 1925). Roots can also be attracted to other roots; for instance, *Fragaria vesca* (wild strawberry) roots are drawn to *Glechoma hederacea* (ground ivy) roots, whereas the ivy roots avoid the strawberry roots (de Kroon, 2007). In addition, root elongation responses also occur: for instance, elongation of *Fragaria chiloensis* (beach strawberry) roots is stimulated upon contact with ground ivy (Semchenko et al., 2007b). Analysis of the overall root biomass of natural grassland systems revealed overyielding, no effect, or even underyielding when mixtures are compared with monocultures (Faget et al., 2013). In crops as well, effects on root growth by neighboring roots are clear, not only intraspecifically, such as for *Glycine max* (soybean) and *Allium cepa* (onion; Raper and Barber, 1970; Baldwin and Tinker, 1972), but also when intercropped. For instance, when certain *Zea mays* (maize) and soybean species are grown together, the roots of each plant tend to keep away from each other and become shallower than those in systems intercropped with their kin. Remarkably, not every maize variety responds in the same manner to the presence of the same soybean species (Fang et al., 2011). Similarly, roots of a *Beta vulgaris* (beet) variety grow faster and deeper than legume roots grown in the same soil, providing a competitive advantage (Tosti and Thorup-Kristensen, 2010). Furthermore, roots can accumulate in the top soil, such as in mixed grassland species (Mamolos et al., 1995). The root density in the top soil of *Acacia saligna* (orange wattle) trees intercropped with *Sorghum bicolor* (sorghum) is also higher than that of monocultures (Lehmann et al., 1998). Intriguingly, root allocations might be influenced by kin recognition, i.e., the ability to discriminate siblings from strangers. In *Cakile edentula* (sea rocket) and *Impatiens pallida* (pale touch-me-not), root allocation is larger and smaller in groups of strangers than of siblings, respectively (Dudley and File, 2007; Murphy and Dudley, 2009). In conclusion, responses (Figure 1A) vary in several experiments, indicating that roots sense the presence of other roots and that identity recognition might be important in altering root growth.

## IDENTITY RECOGNITION AND ITS IMPORTANCE

In plants, identity recognition has been unequivocally demonstrated for several biological processes that affect plant fitness, reproduction, and/or survival. For instance approximately 60% of the angiosperms show self-incompatibility which ultimately serves to prevent self-fertilization (for reviews, see Kitashiba and Nasrallah, 2014; Sawada et al., 2014). Moreover, host recognition systems of parasitic plants (Cardoso et al., 2011) and recognition of potential pathogens basically relies on the ability to discriminate “self” and “non-self” (Sanabria et al., 2008).

As shown above, identity recognition is also of great importance for the outcome of belowground interactions. Self/non-self recognition had first been reported for the desert shrub *Ambrosia dumosa* (burro-weed). Roots of *Ambrosia* stop growing when root systems from other *Ambrosia* plants (i.e., the same plant population) are encountered, seemingly as an avoidance response, but not when roots from the same physiological individual (i.e., self roots) are sensed (Mahall and Callaway, 1991, 1992). In contrast, roots of *Larrea tridentata* (creosote bush), also a desert shrub, tolerate neither other *Larrea* nor *Ambrosia* roots in their proximity (Mahall and Callaway, 1991, 1992). Since these first findings, self/non-self identity recognition has been studied in various species, but no uniform responses are observed. In some species, non-self roots seem to promote root growth traits, whereas root growth is not enhanced by self roots (Table 1). Nonetheless, a clear conclusion could be drawn, namely that to be recognized as self roots, they must be physiologically attached. Detached roots, even when they originate from the same and, thus, genetically identical individual, are recognized as non-self (Mahall and Callaway, 1991; Gruntman and Novoplansky, 2004; Falik et al., 2006; Nord et al., 2011). In contrast, kin recognition or recognition of the same species/population, but not of the same individual would occur via different mechanisms. In *Arabidopsis*, photosensory receptors distinguish between light signals from kin and other neighbors and allow leaf repositioning to decrease light competition (Crepy and Casal, 2014). The distinction between strangers and siblings could also be based on genetic similarity, although both in *Pisum sativum* (pea) and *Buchloe dactyloides* (buffalograss; Falik et al., 2003; Gruntman and Novoplansky, 2004) intermediate responses and phenotypes during self/non-self root experiments hint at certain overlaps.

**Table 1:** **Overview of self/non-self root recognition studies: parameters analyzed, outcome, and used species.**

Parameter	Effect	Species	Reference
Root elongation rate	Decline for non-self roots, no effect for self roots	*Ambrosia dumosa*	Mahall and Callaway (1991)
	Decline for both self and non-self roots	*Larrea tridentata*	Mahall and Callaway (1991)
Root growth (length and/or number)	No effect	*Andropogon gerardii*	Markham and Halwas (2011)
	Reduced for non-self, no effect for self	*Arabidopsis thaliana*	Biedrzycki et al. (2010)
	Fewer and shorter roots toward self	*Buchloe dactyloides*	Gruntman and Novoplansky (2004)
Lateral roots	More and longer lateral roots toward non-self	*Pisum sativum*	Falik et al. (2003)
Root segregation	Roots avoid non-self roots, no effect for self roots	*Arabidopsis thaliana*	Caffaro et al. (2011)
	Spatial segregation for self roots	*Fragaria chiloensis*	Holzapfel and Alpert (2003)
	Attraction for same genotype, avoidance for different genotypes	*Oryza sativa*	Fang et al. (2013)
	No effect	*Fragaria vesca*	Semchenko et al. (2007b)
	Avoidance for self and non-self	*Glechoma hederacea*	Semchenko et al. (2007b)
Root biomass	No effect of neighboring plants	*Avena sativa*	Semchenko et al. (2007a)
	Self-inhibition	*Glycine max*	Gersani et al. (2001)
	Less biomass in presence of self roots	*Trifolium repens*	Falik et al. (2006)
	Overproliferation toward non-self	*Phaseolus varigaris*	Maina et al. (2002)

The impact of root growth inhibition by other plants, whether they are self, strangers, or kin, can be intuitively explained in terms of “space defense” and resource availability. Indeed, inhibition is less demanding than direct competition for the same nutrients in the shared space (for a review, see Schenk et al., 1999). In contrast, root growth overproliferation might maximize the nutrient uptake, but could also affect propagation. In this so-called “tragedy of the commons” that is demonstrated in soybean (Gersani et al., 2001) and *Phaseolus varigaris* (Kenya beans; Maina et al., 2002), the root overproliferation response reduces the reproductive biomass without competitive advantages. However, other studies (Holzapfel and Alpert, 2003; Gruntman and Novoplansky, 2004) do not indicate shoot or reproductive mass changes, so root growth inhibition is not always paralleled by obvious aboveground modifications and could be species specific. Besides biomass alterations, quick physiological responses can be mediated by root identity recognition. In pea, root competition does not affect photosynthesis, although leaf dark respiration is halved, whereas root respiration increases in the vicinity of non-self roots (Meier et al., 2013).

Root overproduction, at the expense of reproductive or shoot biomass, suggests that regulation of the identity recognition can be an important means to increase crop yields. In the cases in which the tragedy of the commons had been observed, isolation of plants from each other could enhance yield (e.g., biomass, seeds, fruits, and flowers) with the same input of water and nutrients (Maina et al., 2002). Moreover, data derived from transcriptomics techniques, only recently applied in the field of root identity recognition, can prove useful. Thus far, differentially expressed gene sets have been reported for intraspecific and interspecific competition of *Arabidopsis thaliana* (thale cress) plants and for *Centaurea maculosa* (spotted knapweed; Broz et al., 2008; Biedrzycki et al., 2011; Masclaux et al., 2012; Schmid et al., 2013). The existence of a core gene set involved in identity recognition, as suggested by Schmid et al. (2013), merits further research. Moreover, identity recognition seems to be evolutionarily conserved because it has been reported already in spermatophytes (Gorelick and Marler, 2014). The molecular biology behind root identity recognition should be tested exhaustively, for example, by employing deep sequencing methods. Biedrzycki et al. (2011) and Schmid et al. (2013) demonstrated that the molecular responses of root and pathogen recognition overlap. Comparison of their datasets with datasets of plant growth promoting rhizobacteria that can accelerate the growth and vegetative phase of plants (Poupin et al., 2013) can be most relevant for crops. The results might have great applications in agricultural practices, in addition to the discovery of the mechanisms responsible for identity recognition that have long been elusive.

## MECHANISMS OF IDENTITY RECOGNITION IN PLANTS

For self-incompatibility, specific ligands are involved (Sawada et al., 2014). Volatile cues from self cuttings of *Artemisia tridentata* (sagebrush) increase herbivore resistance when compared to volatiles from non-self cuttings (Karban and Shiojiri, 2009). In addition, light signals mediate discrimination between kin and neighbors, leading to leaf repositioning which requires auxin biosynthesis (Crepy and Casal, 2014). Regarding root communication (Figure 1B), mediation through electrical signals has been proposed (Schenk et al., 1999). Furthermore, experimental data in pea demonstrate that hormonal rhythms might be implicated (Falik et al., 2003), as corroborated by Gruntman and Novoplansky (2004) who concluded that an unknown physiological mechanism (i.e., electrical or hormonal rhythm) might be responsible for root discrimination in buffalograss. Differential internal oscillatory signals and their resonant amplification would lead to the recognition of a non-self root. Alternatively, perception of neighboring roots has been proposed to be attributed to associated microorganisms and their secreted substances (Steenhoudt and Vanderleyden, 2000). Transcriptomics data have confirmed this hypothesis by the striking overlap of genes associated with plant reactions to neighbors and with responses to pathogens (Biedrzycki et al., 2011; Schmid et al., 2013). Surprisingly, however, in axenic cultures of *Arabidopsis* plants exposed only to root exudates—i.e., the mixture of compounds that are actively secreted or passively released by roots (Bais et al., 2006)—of strangers, the induction of lateral root formation is higher than that after exposure to sibling exudates. This observation suggests that a soluble chemical, originating from root exudation, might be responsible for identity recognition (Biedrzycki and Bais, 2010a,b; Biedrzycki et al., 2010). By means of the root secretion inhibitor sodium orthovanadate, which blocks active root secretion of several phenolic compounds in *Arabidopsis*, seedlings no longer recognize strangers, implying that active secretion by roots is required for kin recognition (Biedrzycki et al., 2010). However, self/non-self recognition is not influenced by the secretion inhibitor, confirming that two separate identity recognition mechanisms exist. Caffaro et al. (2011) demonstrated that self and non-self exudates similarly reduce root growth, but that addition of activated charcoal, specifically reverses the effect of self roots on root growth, indicating that reduced amounts of secondary metabolites in the medium affected self/non-self recognition. Consistently, root proximity is important for interaction responses in *Oryza sativa* (rice). Exclusion of aerial interactions by shoot separation experiments hinted at the induction of interactions by root exudates that diffuse into the medium rather than by physical contact (Fang et al., 2013). In *Deschampsia cespitosa* (tufted-hair grass), root exudates have also been demonstrated as cues of neighbor identity that control root mass and morphology (Semchenko et al., 2014). Noteworthy, root-object recognition might occur via allelopathic root exudates, as shown in pea (Falik et al., 2005), but, according to recent evidence in rice, could also be mediated via different processes that require physical contact of the root tip with the obstacle (Fang et al., 2013). Unraveling the exact nature of the signals that trigger identity recognition would be a gigantic leap forward in root–root interaction studies.

## SELF/NON-SELF RECOGNITION IN ROOTS: PITFALLS

The interpretation of some of the experiments concerning self/non-self recognition remains somewhat controversial (for a review, see Chen et al., 2012). As already mentioned above (see also Table 1), different species are used to study self/non-self identity recognition in plants, making it difficult to draw clear conclusions due to likely species-specific and genotype-specific effects (Fang et al., 2011). Moreover, the root growth strategies of the species under study might influence the outcome of the experiments. For instance, in the strawberry/ivy experiments, strawberries grow clonally and always spread widely within plant communities, which may well affect whether a neighboring root will be attracted or avoided (de Kroon, 2007; Faget et al., 2013).

Moreover, several parameters have been analyzed during root recognition research, such as root biomass, adaptation of root architecture/morphology, and root length (Table 1). Most studies focus on root biomass, but root architecture may well be the primary and quickest response that does not necessarily impose an altered photosynthate allocation when compared to mere root growth, as demonstrated in *Phaseolus vulgaris* (common bean; Nord et al., 2011).

Split-root experimental systems have been used to study self/non-self recognition in root (Gersani et al., 2001; Maina et al., 2002; O’Brien et al., 2005), but the effects of pot volume and nutrient levels, which are important factors determining root growth, are difficult to correct and will influence responses to strangers and/or identity recognition. Indeed, several results can be rationalized as responses to soil volume (Schenk, 2006; Hess and de Kroon, 2007; Markham and Halwas, 2011). Nonetheless, other experiments have unequivocally demonstrated root mass changes that depend solely on the identity of the interacting root, as, for instance, in buffalograss (Gruntman and Novoplansky, 2004). In addition, plants would react to available resources rather than to the presence of a neighbor that will, while growing, deplete the same soil zone from soil nutrients (Semchenko et al., 2007a). Indeed, roots grow preferentially where supplies are most accessible (Gersani et al., 1998; Hodge, 2009), the probable reason for avoidance of other root systems. Therefore, nutrient levels and detection of the presence of other root systems are often confounded as well (O’Brien et al., 2005; Klemens, 2008; Fang et al., 2011). For example, common bean plants will change their root system architecture and produce fewer roots in soil patches that are occupied by neighboring roots (Nord et al., 2011), possibly in relation to the phosphorus concentration in the soil, which is nearly immobile and influences the developmental plasticity of roots (e.g., Borch et al., 1999; reviewed in Ticconi and Abel, 2004). Nonetheless, transcriptomics analysis of *Arabidopsis* in the presence or absence of competing *Hieracium pilosella* (mouse-ear hawkweed) clearly indicate that sensing neighboring roots occurs before resource depletion is discovered (Schmid et al., 2013).

In addition to nutrients and soil volume, water availability must be considered as well. In *Ambrosia dumosa*, intraspecific water competition is thought to be the reason for growth reduction when self roots are recognized (Mahall and Callaway, 1992). Hence, local changes in the microclimate should also be taken into account; for instance, root temperature gradients affect root productivity and lead to top soil accumulation (Füllner et al., 2012). Indeed, these problems have been recognized and highly controlled experiments have been set up in which plants are grown in preconditioned liquid media (Biedrzycki et al., 2010; Caffaro et al., 2011). Nevertheless, because these systems remain artificial, their relevance in natural soil systems can be questioned. Alternatively, the use of clonal ramet pairs (Holzapfel and Alpert, 2003; Gruntman and Novoplansky, 2004; Semchenko et al., 2007b) was suggested to circumvent the above mentioned problems. However, although pot volume and nutrient levels could indeed be kept constant, disconnected ramets would still be considered to access only half the amount of nutrients (i.e., two one-root system plants) as compared to connected plants (a single two-root system plant) that have access to the full amount of nutrients. These approaches have thus been criticized as well (Hess and de Kroon, 2007).

Moreover, quantification of belowground interactions is difficult, certainly at the level of the individual root. Although beyond the scope of this minireview, recent advances in imaging technology might be helpful. A transparent gel system is now developed that allows imaging and three-dimensional reconstruction to quantitatively assess root growth parameters during interaction studies (Fang et al., 2013). Likewise, fluorescent markers and horizontal minirhizotrons imaging systems (Faget et al., 2009, 2012) have proven successful to study maize, *Lolium multiflorum* (Italian ryegrass), and soybean interactions. As a drawback, genetically modified plants are required that, hence, hamper ecological applications. These non-destructive technologies outcompete the mere analysis of root biomass and are promising alternatives for root–root interaction and root identity recognition studies.

In conclusion, exciting advances in the field of self/non-self recognition of roots have been made over the recent years. New imaging technologies will not only aid to analyze the root response in a non-destructive way, but will also allow kinetics studies that will help to understand the mechanisms of root identity recognition and to avoid the confusion of the effects of root interactions with those of nutrients and root volume. The identification of a core set of genes involved in neighbor detection merits further research and functional analyses. New and high-resolution chemical analysis techniques, besides state-of-the-art techniques used to measure electrical signals *in planta*, as well as molecular biological approaches should be utilized to clarify root–root identity recognition. The obtained results can have an enormous impact on the research in plant biology and development as well as on the agricultural and ecological research fields and practice.

### Conflict of Interest Statement

The author declares that the research was conducted in the absence of any commercial or financial relationships that could be construed as a potential conflict of interest.
